# Surgery for severe aortic stenosis with low transvalvular gradient and poor left ventricular function – a single centre experience and review of the literature

**DOI:** 10.1186/1749-8090-2-9

**Published:** 2007-01-31

**Authors:** Andreas Borowski, Ali Ghodsizad, Ilja Vchivkov, Emmeran Gams

**Affiliations:** 1 Department of Thoracic and Cardiovascular Surgery, University of Düsseldorf, Germany

## Abstract

**Background:**

A retrospective comparative study was designed to determine whether the transvalvular gradient has a predictive value in the assessment of operative outcome in patients with severe aortic stenosis and poor left ventricular function.

**Methods:**

From a surgical database, a series of 30 consecutive patients, who underwent isolated aortic valve replacement for severe aortic stenosis with depressed left ventricular (LV) function (EF < 40%), were enrolled in the study and divided into two groups according to the mean transvalvular gradient (TVG): LG(low gradient)-Group < 40 mmHg (n = 13), and HG(high gradient)-Group > 40 mmHg (n = 17). Both groups were then comparatively assessed with respect to perioperative organ functions and mortality.

**Results:**

Both groups were well matched with respect to the preoperative clinical status. LG-Group had a larger aortic valve area, higher LVEDP, larger LVESD and LVEDD, and higher mean pulmonary pressures. The immediate postoperative outcome, hospital morbidity and mortality did not differ significantly among the groups.

**Conclusion:**

In patients with severe aortic stenosis and poor LV function, the mean transvalvular gradient, although corresponds to reduced LV performance, has a limited prognostic value in the assessment of surgical outcome. Generally, operating on this select group of patients is safe.

## Background

In patients with severe aortic stenosis (AS) scheduled for aortic valve replacement (AVR), the operative risk as well as the late postoperative outcome increases with the onset of LV systolic and diastolic dysfunction [1,2]. As the results of AVR in patients with severely reduced LV function and low TVG are uncertain, this patient subset still remains the most controversial in terms of decision making for surgery [3].

Few data are available on the immediate postoperative and long-term outcome in patients with isolated severe AS and poor LV function, especially in regard to the severity of TVG.

We tested the hypothesis that AVR in patients with isolated severe AS and poor LV function, the mean TVG has no impact on immediate postoperative outcome. Indications for surgery in the light of current opinions published in the literature are discussed.

## Methods

From our surgical database including a total number of 1117 patients who between 1992 and 2002 underwent AVR, a series of 30 consecutive patients with severe aortic stenosis associated with depressed LV function, was selected for the study.

Inclusion criteria were: LVEF < 40 %, and aortic valve area (AVA) of < 1 cm^2 ^(additionally confirmed at surgery). Patients with associated coronary heart disease (CHD) or concomitant valve procedure or with significant aortic valve regurgitation were excluded from the study. All study patients were operated on under conditions of extracorporeal circulation (ECC), using moderate hypothermia, and Bretschneider's cardioplegic solution for myocardial protection.

The study cohort was divided into two groups according to the level of the mean TVG: LG-Group < 40 mmHg, and HG-Group > 40 mmHg. Due to a relatively high percentage of patients with atrial fibrillation, mean gradients were used to define study groups [4]. The pre-, peri- and postoperative data of both groups were comparatively analysed.

The preoperative data collection included: demographic data, clinical symptoms with special regard to respiratory insufficiency and NYHA functional class, rhythm disturbances, admission-to-surgery interval, degree of urgency, echocardiographic examination, cardiac catheterization, organ functions, and need for preoperative emergency balloon valvuloplasty. Respiratory insufficiency was defined as the need for non-invasive (face mask) or invasive mechanical ventilation.

As relevant perioperative data, type and size of the prosthetic valve implanted, the effective orifice area index (calculated as EOA divided by body surface area, EOA/BSA=EOA-I), ischemia- and ECC-time, catecholamine and/or mechanical circulatory support were considered.

The postoperative data comprised assessment of organ functions, catecholamine and mechanical circulatory support, mechanical ventilation time, duration of stay at intensive care unit, and hospital mortality and morbidity. The inotropic support was defined as high, when epinephrine with a dosage of > 6 μg/kg b.w./min, moderate with 4–6 μg/kg b.w./min, and low with < 4 μg/kg b.w./min, for at least 48 h, was administered.

## Results

The demographic and preoperative clinical data of both patient groups are presented in Table 1. With regard to preoperative organ finctions, 3 patients in LG-Group and 5 patients in HG-Group had increased creatinine level (1.6–2.8 mg/dl vs 1.7–2.4 mg/dl). In 2 patients in LG-Group and 6 patients in HG-Group, increased liver transaminase level with GOT values between 35–280 U/l and GPT values between 90–325 U/l, respectively, were observed.

**Table 1:** Preoperative data

	LG-Group n = 13	HG-Group n = 17
f/m ratio (n)	2:11	8:9
Age (y, mean, range)	65 (43–77)	69 (33–86)
Symptoms:		
Dyspnoea	11 pts (85%)	12 pts (70%)
Respiratory insufficiency	5 pts (38%)	6 pts (35%)
Syncope	2 pts (15%)	1 pt (6%)
Cardiogenic shock	2 pts (15%)	2 pts (11%)
AF	4 pts (30%)	4 pts (23%)
Balloon valvuloplasty	0 pt	2 pts (11%)
NYHA class III/IV	9 pts (69%)	12 pts (70%)
Priority:		
Emergent	2 pts (15%)	3 pts (17%)
Admission-to-surgery		
interval (d, mean, range)	33.2 (5–85)	11.5 (1–60)
EuroSCORE (mean, range)		
logistic (%)	22.3 (2.77–59.48)	21.03 (3.13–84.53)
additive	9.8 (3–16)	9.4 (5–19)

*AF *atrial fibrillation, *d *days, *f/m *female/male, *h *hours, *HG *high gradient, *LG *low gradient

The admission-to-surgery time interval was considerably longer in LG-patient group, whereas in 2 patients in LG-Group und 3 patients in HG-Group, due to a rapid deterioration of circulatory conditions, surgery was performed on an emergency basis. Two of 5 patients with respiratory insufficiency in LG-Group and one of 6 patients in HG-Group required mechanical ventilation. The preoperative hemodynamic situation with echocardiographic and cardiac catheterization data for both patient groups are summarized in Table 2.

**Table 2:** Preoperative echocardiographic and cardiac catheterization data

Parameters	LG-Group	HG-Group
TVG (mmHg)	35 (25–40)	90 (60–120)
LVEF (%)	30 (12–40)	25 (15–40)
AVA (cm^2^)	0.7 (0.6–0.9)	0.5 (0.3–0.6)
LVEDP (mmHg)	40 (20–44)	24 (18–32)
LVESD (mm)	50 (39–64)	44 (24–50)
LVEDD (mm)	70 (52–74)	60 (46–70)
PAP (mmHg)	44 (25–80)	30 (18–90)
C.I. (L/min/m^2^)	1.8 (1.7–2.2)	2.0 (1.8–2.5)

*AVA *aortic valve area, *C.I*. Cardiac index, *HG *high gradient, *LG *low gradient, *LVEDD *left ventricular end-diastolic diameter, *LVEDP *left ventricular end-diastolic pressure, *LVEF *left ventricular ejection fraction, *LVESD *left ventricular end-systolic diameter, PAP pulmonary artery pressure, *TVG *mean aortic transvalvular gradient

Data are given as mean values with ranges in brackets

The median size of implanted valves was 25 mm and 23 mm for LG- and HG-Group, respectively. In LG-Group, 10 mechanical valves (9 SJM HP Type, 1 SJM Regent, St. Jude Inc., St.Paul, MN, USA) and 3 biological valves (Baxter Perimount, Baxter Healthcare Corporation, Irvine, CA, USA) were implanted. In HG-Group, 11 patients received mechanical (SJM HP), and 6 patients biological (Baxter Perimount) valve prosthesis.

The EOA-I was calculated 1.48 ± 0.28, median 1.41, range 0.94–1.93 for LG-Group, and 1.39 ± 0.27, median 1.43, range 0.88–1.8 for HG-Group.

The mean myocardial ischemia time (aortic cross-clamp) for LG- and HG-Group were with 65 and 67 minutes, and the mean ECC duration 102 and 123 minutes, respectively. The immediate postoperative catecholamine support was comparable among the groups: in LG-Group, 4 patients needed high, 5 patients moderate, and in HG-Group, 5 patients high, and 5 patients moderate inotropic support; 2 patients in LG-Group and 3 patients in HG-Group were implanted with intraaortic ballon pump for mechanical circulatory support.

The relevant postoperative data are summarized in Table 3.

**Table 3:** Postoperative data

	LG-Group	HG-Group
Mechanical ventilation (h, mean, range)	13 (6–48)	22 (6–72)
CVVH	2 pts (15%)	4 pts (23%)
Rethoracotomy for bleeding	3 pts (23%)	2 pts (11%)
Permanent a-v block III°	1 pt (7%)	1 pt (6%)
IABP	2 pts (15%)	3 pts (17%)
ICU stay (d, mean, range)	3.7 (1–12)	5.4 (2–19)
Mortality	1 pt (7%)	1 pt (6%)

*CVVH *continuous veno-venous hemofiltration, *d *days, *h *hours, *HG *high gradient, *IABP *intraaortic balloon pump, *ICU *intensive care unit, *LG *low gradient

## Discussion

Patients with severe LV dysfunction, particularly those with low TVG, although account for only 5–10% of patients with AS, represent most controversial subset with a difficult management decision [3]. The underlying management problems focus on the operative mortality and potential for recovery of LV function.

In recent studies, perioperative mortality in patients with AS and LV dysfunction is reported to vary between 3% and 62%, and the results are strongly dependent on underlying hemodynamic conditions and/or concomitant procedures [5-7]; e.g. patients with severe AS and associated CHD are considered as a subset with increased perioperative risk [8,9].

The contractile reserve (CR) seems to play an essential role for operative outcome.

Low perioperative mortality rates of 5% and 3% were published by Monin [5,10] and Powell [8], respectively, in patients with isolated AS and LV dysfunction and with dobutamine proven CR. Schwammenthal [7], based on Dobutamine Stress Echocardiography (DSE), in patients with AS and severe LV dysfunction, observed good surgical outcome when the clinical decision was concordant with the results of DSE.

In attempt to define the criteria for selecting the patients who will benefit from AVR, Monin [5], based on the results of a multicentre study, assessed the prognostic value of dobutamine stress hemodynamic data in the setting of low-gradient AS: predictors for operative mortality were the lack of CR and a mean TVG < 20 mmHg (mortality 5% vs 32%). The author finally concluded that predictive value of the lack of CR in terms of postoperative changes is still not known. Quere [11] observed that absence of CR is related to high operative mortality but does not predict the absence of LVEF recovery, suggesting that surgery should not be contraindicated on the basis of absence of CR alone.

A summarized review of surgical outcome in recent series is depicted in Table 4.

**Table 4:** Outcome in recent series

Author	n	EOA/EF	PtsCHD				Mortality		
				Total	-CHD	+CR	-CR	+CR +CHD	-CR +CHD
Connolly [3]	52	< 0.9/< 35	62%	21%					
Monin [5]*	136	< 0.8/< 35	40%	14%		5%	32%	11%	62%
Powell [8]	55	< 0.75/< 30	36%	18%	3%				
Tarantini [9]	52	< 1.0/< 35	30%	8%					
Monin [10]	30	< 0.8/< 30	43%	16%		8%	50%		
Nishimura [15]	21	< 1.0/< 40	70%	14%		14%			

*EOA/EF *effective orifice area (cm^2^)/ejection fraction (%), *PtsCHD *proportion of patients with coronary heart disease, *Mortality:-CHD *patients without coronary heart disease, *+CHD *patients with coronary heart disease, -*CR *patients with no contraction reserve, +*CR *patients with contraction reserve, *periop *perioperative, *n *number of patients, * multicentre study

Although the preoperative LV dysfunction represents increased risk for perioperative mortality, generally, LV performance recovers after AVR [12,13]. Brogan [14] reported, that some of the patients with severe AS and severe LV dysfunction, and preoperative NYHA functional class III/IV were found to be in class I after 8–36 months postoperatively. Nishimura [15] suggested that, despite comparable poor preoperative cardiac performance, a very different recovery potential of the failing heart is possible. Rothenburger [16] reported at a follow-up of 1 year after AVR, an improvement of EF of up to 40%. In recent studies [8,10,15], a postoperative increase of EF at an avarage of up to 100% during a follow-up time of 3–5 years was also observed. It should be stressed however, that in the most recent relevant studies, all patient groups with severe AS and severe LV dysfunction who underwent AVR were relatively small, and had concomitant CABG procedure or no informations about the concomitant procedures were given [5,7,10,15].

### Limitations of the study

In none of our patients a DSE was performed.

For comparison, two patient cohorts with low- and high TVG, both with severely depressed LV function were selected. Reduced EF is considered to be related to excessively high wall stress due to insufficient hypertrophy and not irreversibly impaired contractility [2,17]. If so, the ability to develop/increase the TVG despite severe LV dysfunction can be interpret as a available CR. And congruently, with comparable mean LVEF for both groups in our patient collective, the HG-patient group had considerably smaller LVEDD and LVESD, lower LVEDP- and PAP-values. One can therefore assume that patients in LG group had a lower CR when compared to HG-Group. Although not statistically proven, our observations are contradictory to those published by Monin [18], who stated that in the setting of poor LV-function, the decrease of TVG has an independent prognostic value for operative risk.

## Conclusion

Despite higher degree of LV dysfunction, our patients with low gradients had comparable outcome to those with high gradients, suggesting that operating on this select group of patients is a safe procedure. However, in high risk patients, preoperative risk assessment with estimation of myocardial contraction reserve, adequate judgment of severity of aortic stenosis, consideration of co-morbidity and need for concomitant procedures are mandatory to avoid operating on patients who will not benefit from surgery [5,7-9,14,19].
